# Effect of initial moisture content on the in-vessel composting under air pressure of organic fraction of municipal solid waste in Morocco

**DOI:** 10.1186/1735-2746-10-3

**Published:** 2013-01-03

**Authors:** Abdelhadi Makan, Omar Assobhei, Mohammed Mountadar

**Affiliations:** 1 Water and Environment Laboratory, Chemistry Department, Faculty of Science, University Chouaib Doukkali, P.O. Box 20, El Jadida 24000, Morocco; 2 BIOMARE Laboratory, Biology Department, Faculty of Science, University Chouaib Doukkali, P.O. Box 20, El Jadida, 24000, Morocco

**Keywords:** Composting, Moisture content, Municipal solid waste, Air pressure, Bioreactor

## Abstract

This study aimed to evaluate the effect of initial moisture content on the in-vessel composting under air pressure of organic fraction of municipal solid waste in Morocco in terms of internal temperature, produced gases quantity, organic matter conversion rate, and the quality of the final composts.

For this purpose, in-vessel bioreactor was designed and used to evaluate both appropriate initial air pressure and appropriate initial moisture content for the composting process. Moreover, 5 experiments were carried out within initial moisture content of 55%, 65%, 70%, 75% and 85%. The initial air pressure and the initial moisture content of the mixture showed a significant effect on the aerobic composting. The experimental results demonstrated that for composting organic waste, relatively high moisture contents are better at achieving higher temperatures and retaining them for longer times.

This study suggested that an initial moisture content of around 75%, under 0.6 bar, can be considered as being suitable for efficient composting of organic fraction of municipal solid waste. These last conditions, allowed maximum value of temperature and final composting product with good physicochemical properties as well as higher organic matter degradation and higher gas production. Moreover, final compost obtained showed good maturity levels and can be used for agricultural applications.

## Introduction

In Morocco, most municipal solid wastes (MSW) are disposed-off in landfills. The Department of Environment of Morocco reported in 2001 that MSW contains organic content in the range of 50 - 70%. Food scraps and garden waste are the major components of organic waste in MSW but the final composition of organic fraction of municipal solid waste (OFMSW) varies with seasons and consumption types. In general, main Moroccan consumption of organic products consists of vegetables, fruits and tea products (85%) with an average annual consumption of 5,841,440 tons, 2,076,946 tons and 138,740 tons respectively [1]. Thus, the average annual consumption per person is about 167 kg of vegetables, 60 kg of fruit and 4 kg of tea products, while means total consumption of these three products is 231 kg/habitant/year. Furthermore, the decomposition of OFMSW under anaerobic conditions in landfills produces carbon dioxide and methane. Methane, a greenhouse gas 21 times more potent than carbon dioxide, is considered to be a significant contributor to global warming; thus the control of methane emissions from landfills is of great importance [2]. Bogner & Matthews [3] reported that global methane emissions from landfills amount to 16–57 Tg CH_4_ yr^-1^.

Composting has long been recognized as an environmentally acceptable method for treating industrial and agricultural organic wastes [4]. It is a natural process by which micro-organisms decompose organic matter into simpler nutrients. Aerobic composting is the process where decomposition takes place in the presence of oxygen. As the quickest way to produce high quality compost, aerobic composting is a widely accepted way of stabilizing organic wastes and converting them to a usable, and value added compost product [5]. Keener *et al*. [6] enumerated more than 20 factors which affect the decomposition of organic matter in the composting process. Temperature, moisture content, oxygen concentration in the airspace, and C/N ratio are generally recognized as the primary factors affecting the composting process [5-7]. In a typical completely mixed organic waste composting process these factors are controlled by varying ingredient mix ratios, aeration, turning frequency, and occasionally by moisture addition [8].

Moisture content affects microbial activity, as well as the physical structure, in the composting process, and thus has a central influence on the biodegradation of organic materials. Because it is relatively easy to measure, moisture content often serves as a proxy for other critical factors such as water availability, which limits microbial activity in the low moisture range. Very low moisture content values would cause early dehydration during composting, which will arrest the biological process, thus giving physically stable but biologically unstable composts [9]. On the other hand, high moisture may produce anaerobic conditions from water logging, which will prevent and halt the ongoing composting activities [10,11]. Also, high moisture content affects particle aggregation, matrix porosity, air-filled porosity, and matrix gas permeability, all of which can limit transport of essential oxygen into the composting zone where carcass decomposition occurs [12-14]. Because of its importance to the composting process, the effect of moisture content on the decomposition rate has been investigated by many researchers. Previously reported optimum moisture contents for composting range from 25% to 80% on a wet basis (w.b.), with generally recommended values in the 50% to 70% range [5,15,16]. As is evident from this relatively wide range of reported values, there is no universally applicable optimum moisture content for composting materials. This is because each material has unique physical, chemical and biological characteristics, and these affect the relationship between moisture content and its corollary factors water availability, particle size, porosity, and permeability.

Since the composting process is very intricate, it is not easy to estimate the effect of a single factor on the rate of organic matter decomposition. However, to find the best conditions for an effective composting of OFMSW, it is preferable to study separately the influence of each factor. In this study, we investigated two complementary phases which are preliminary study and main study. The preliminary study aims to evaluate the effect of initial air pressure on the in-vessel composting of OFMSW in Morocco, in order to determine the optimum initial pressure (P_op_) that allows efficient composting materials. The main study phase aims to investigate the influence of initial moisture content (MC) on composting of OFMSW under P_op_ pressure.

## Materials and methods

### Composition of OFMSW

Sorting followed by a statistical study is the classical method used for determining the composition of a mixed waste. Furthermore, it is very difficult to adopt this method for organic waste. Organic waste is inseparable either in the landfill or in the same bin. They often have the same color after oxidation. That is why we moved towards an approximate method based on the average consumption of organic products in the country.

Based on the Moroccan consumption of organic products data mentioned in the introduction, experimental results on representative quantities of organic products which showed that vegetables, fruits and tea products generate respectively 17%, 30% and 160% of waste, and population in 2006 (34,859,364), the final composition of the OFMSW was 37% of vegetable waste, 35% of fruit waste, 13% of tea products waste and 15% of other wastes.

### Description of the bioreactor (design)

**Figure 1:**
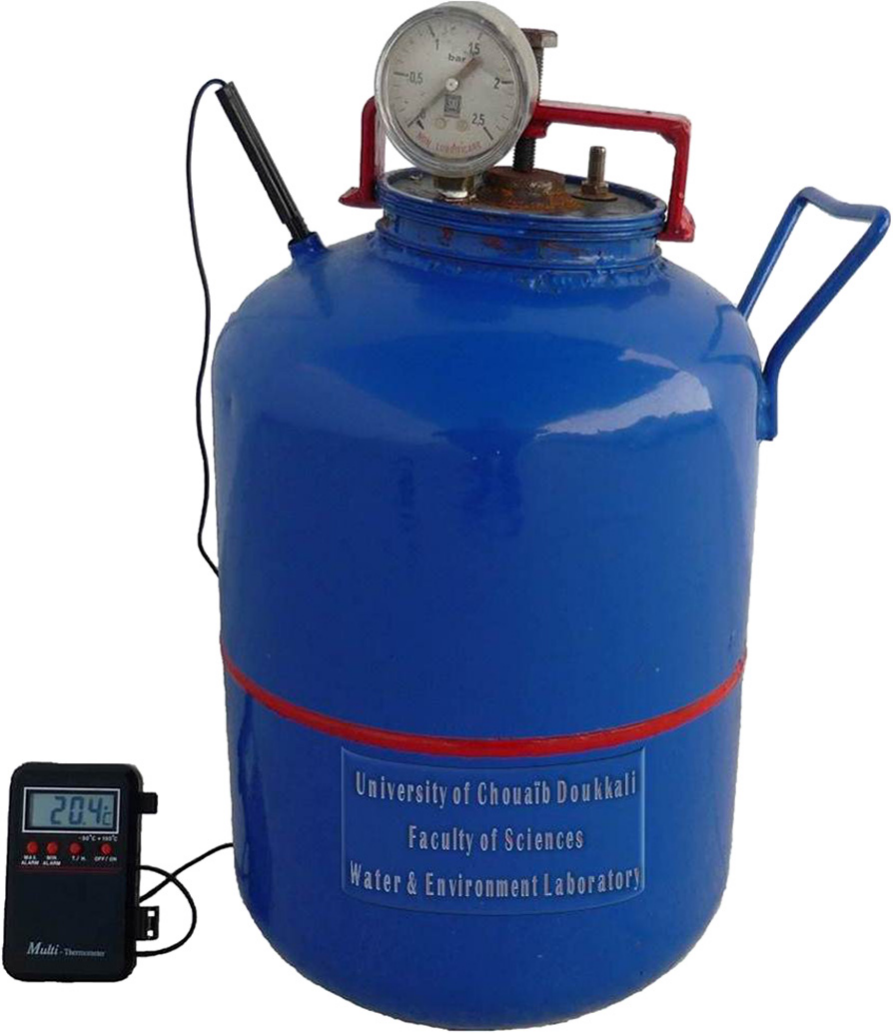
**Laboratory-scale bioreactor.**

The Figure 1 shows the laboratory-scale bioreactor which was specifically designed and used for waste composting in this study. The bioreactor is a vertical metallic cylinder of 15 liters. Metal used is in steel covered by an anticorrosive paint. The metal thickness is 1 mm. The bioreactor is designed with an opening in the upper face for waste introduction. The lid of the upper surface contains a valve to inject and remove air, a manometer to monitor pressure inside the bioreactor and a copper tube. A Multi-thermometer is used to monitor temperature inside the compost. Sealing is ensured by a rubber gasket.

### Waste preparation and experimental design

A representative waste has been prepared based on composition established in section “Composition of OFMSW”. Each species was separately weighed, manually hashed into small pieces of 2 – 5 mm length and added to mixture. The obtained waste was then transferred into plastic boxes and mixed for 30 minutes to achieve materials homogenization. The adjustment of the initial MC was accomplished either by adding required amount of water or by drying the mixture, under sun rays, if there is moisture excess.

A sample of 5 kg of prepared waste was weighed and inserted into the bioreactor. After closing the bioreactor, air was injected through the valve. An air compressor supplies the bioreactor until initial pressure desired. Feeding the bioreactor was made every day. The formed gases during the degradation process were evacuated to the end of the day. Then, the bioreactor was fed a second time and so on until compost stabilization. At outlet, the gas mixture passed through a gas-washing bottle with 1 M sodium hydroxide in order to remove carbon dioxide. The composting process took about 10 days after stabilization. The final compost was then removed from the bioreactor and required indicator values of the process were measured.

The investigation consisted of two complementary phases. The preliminary study was to vary the initial air pressure and keep the initial MC and initial C/N ratio invariable (average MC of MSW in Azemmour: 70% and C/N ratio: 26). Thus, five experiments were conducted to evaluate the initial air pressure effect (0.2, 0.4, 0.6, 0.8 and 1 bar) on the composting process. The second phase which is the main study of this work was to use the optimal value of the initial air pressure (P_op_) determined in the first phase, keeping the C/N ratio invariable and to vary the initial MC. However, five experiments were conducted to evaluate the initial moisture content effect (55%, 65%, 70%, 75% and 85%) on the composting process under the optimum initial air pressure already determined. Some of physicochemical properties of initial mixture were determined and the values were respected for the reproducibility of the experiment. Table 1 shows the average of triplicates values for selected physicochemical properties of initial mixture.

**Table 1:** selected physicochemical properties of initial mixture for different experiments

**Experimental design**		**Process index**			**Physicochemical properties of initial mixture**					
**Phase**	**Experiment**	**P(bar)**	**MC(%)**	**C/N ratio**	**pH**	**EC mS/cm**	**OM(%)**	**TOC(%)**	**TKN(%)**	**NH**_**4**_^**+**^**mg/kg**
1	1	0.2	70	26	6.38	7.2	92.4	46.2	1.82	515
	2	0.4	70	26	″	″	″	″	″	″
	3	0.6	70	26	″	″	″	″	″	″
	4	0.8	70	26	″	″	″	″	″	″
	5	1	70	26	″	″	″	″	″	″
2	1	P_op_	85	26	6.82	7.0	92.8	46.5	1.86	524
	2	P_op_	75	26	6.80	7.2	″	″	″	″
	3	P_op_	70	26	6.68	7.4	″	″	″	″
	4	P_op_	65	26	6.62	7.4	″	″	″	″
	5	P_op_	55	26	6.44	7.5	″	″	″	″

### Analytical methods

Volumetric titration was used to determine carbon dioxide content. An aliquot volume of sodium hydroxide solution was titrated against a standard solution of 1 M hydrochloric acid with phenolphthalein used as an indicator. The gas-washing bottle was changed daily for determination of produced gases. The moisture content of sample was measured after drying at 105°C for overnight. The dried sample was ground and then used in analysis. The organic matter was calculated from the ash after igniting a sample of 20 g dry weight at 550°C for 6 h. The water-soluble extract was prepared by the following procedure: 10 g of sample were first mixed with 100 ml of deionized water, then shaken for 2 h, and centrifuged at 3000 rpm. The supernatant was then filtered through 0.45 μm membrane filters. TOC (total organic carbon) and TKN (total Kjeldahl nitrogen) were measured by the Walkley Black method and semi-micro Kjeldahl method, respectively [17,18]. pH and electrical conductivity were measured in the condition of solid-to-water mixture (weight:volume = 1 : 10). Values were directly read on pH-522 WTW meter and EC-214 conductivity meter, respectively. Ammonium nitrogen, *NH*_4_^+^ − *N*, was measured by the spectrophotometry of salicylic acid and sodium hypochlorite [19]. Temperature in the bioreactor was measured using a Multi-stem digital thermometer (ST-9283B). Evolution of internal pressure was followed using a manometer (0–2.5 bar) to measure gas pressures.

The loss (or conversion) of organic matter, k, was calculated from the initial and final organic matter contents, according to the following equation [5,20,21]:

(1) $$k=\frac{\left[OM_{m}\left(\%\right)-OM_{p}\left(\%\right)\right]100}{OM_{m}\left(\%\right)\left[100-OM_{p}\left(\%\right)\right]}$$

where OM_m_ is the organic matter content at the beginning of the process (%); and OM_p_ is the organic matter content at the end of the process (%).

All analyses were triplicated in order to ensure reproducibility and representativeness of the sample.

## Results

### Preliminary study (initial Air pressure effect)

#### Evolution of internal pressure

**Figure 2:**
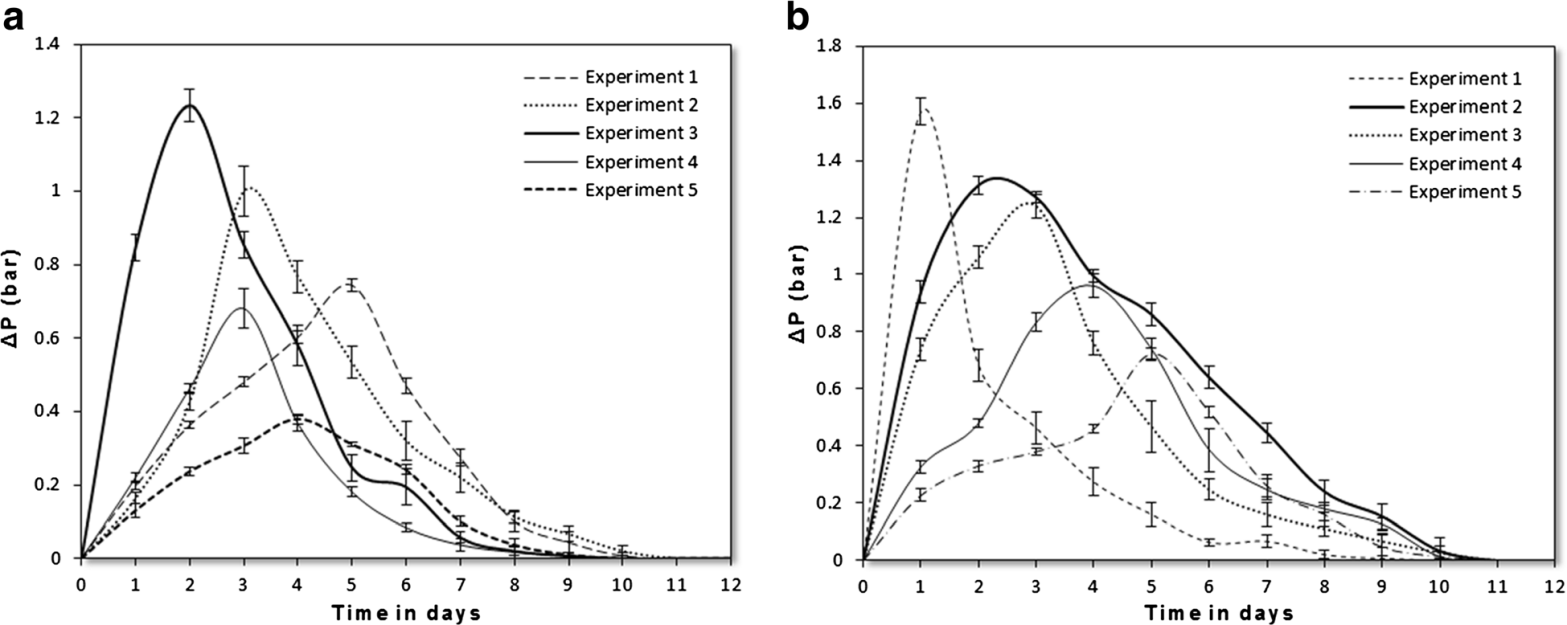
**Evolution of internal pressure; (a) preliminary study, (b) main study.**

The composting process took about 10 days and the required indicator values of the process were measured. Figure 2a shows the evolution of pressure change, ΔP, inside the bioreactor for the five experiments. In the first experiment, pressure change, ΔP, reached its maximum value towards the fifth day of composting with a ΔP of 0.72 bar. The pressure variations in the second and fourth experiment reached their maximum values at the third day of composting process with a ΔP of 0.9 and 0.6 bar, respectively. The greatest ΔP value is given by the third experiment at the second day of composting with a variation of 1 bar. Because of the air oxygen abundance in the bioreactor, this experiment allows better organic matter degradation by micro-organisms and production of gases that are responsible of internal pressure increase. This was consistent with results reported by Makan & Mountadar [22]. Then, the fifth experiment has the lowest value of ΔP and that is at the fourth day of composting with 0.38 bar.

#### Temperature profile

**Figure 3:**
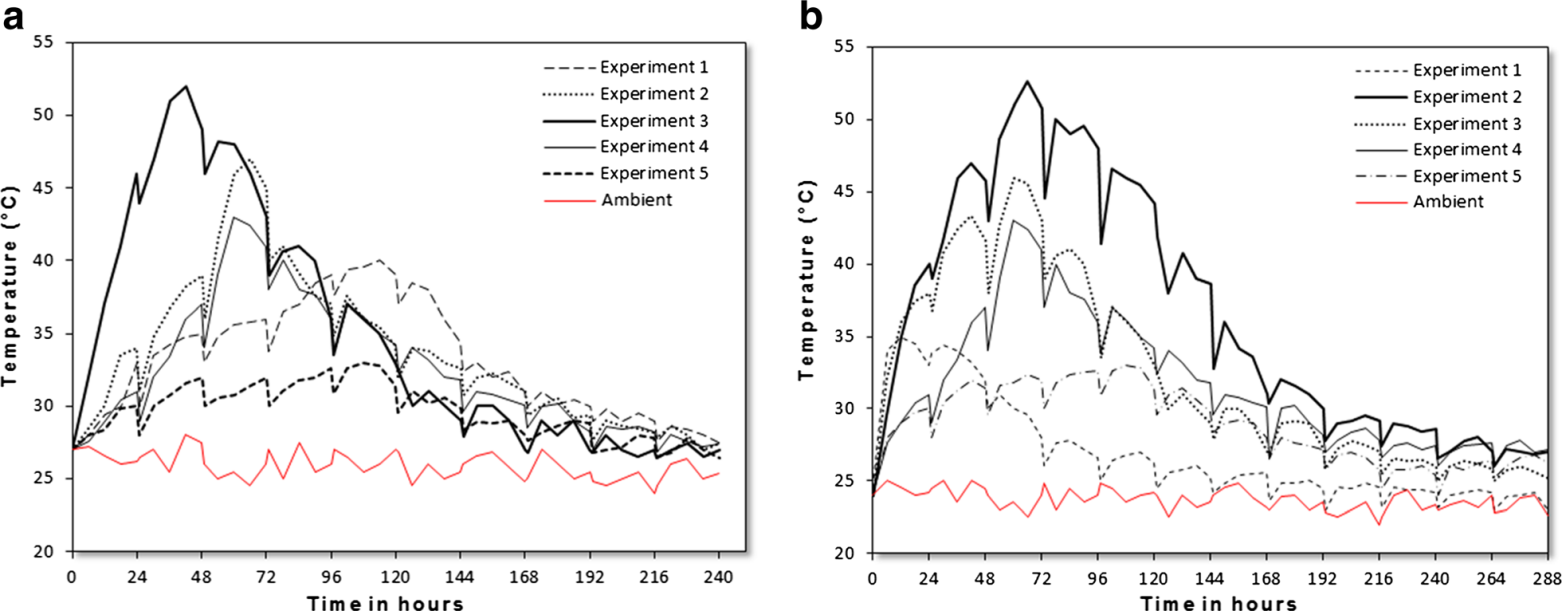
**Change of temperature; (a) preliminary study, (b) main study.**

The temperature has been widely recognized as one of the most important parameters in the composting process, and the variation of temperature has been reported to correlate with microbial activities [23]. Figure 3a shows the average temperature profile at different initial pressures during the composting process. It reaches the maximum value of 43°C, 47°C, 52°C, 40°C and 33°C in first, second, third, fourth and fifth experiment respectively after 5, 3, 2, 3 and 5 days. However, there was no significant difference in temperature regime among all composting experiments after 9 days and we can consider that the compost is stabilized. Compared to all experiments, experiment 3 is most successful because temperature has reached maximum value and this is consistent with the optimal activity of micro-organisms.

#### Evolution of carbon dioxide

**Figure 4:**
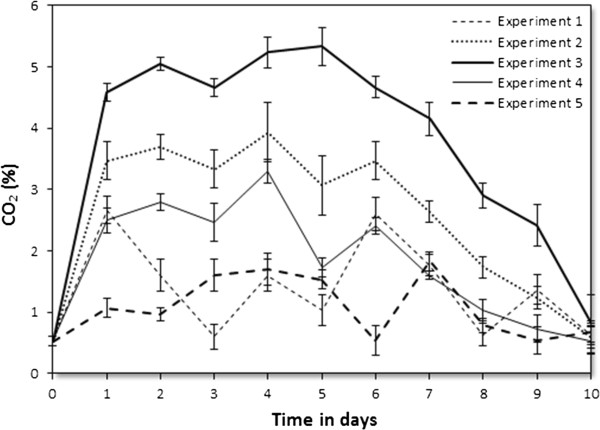
**CO**_**2**_**rate changes in different experiments during the preliminary study.**

CO_2_ production is caused by mineralization of the compost′s organic matter [24]. The results of the carbon dioxide changes for the five experiments in the first phase are shown in Figure 4. The rate of carbon dioxide produced in all experiments increased proportionally to activity of the micro-organisms during the process. The highest rate of carbon dioxide was produced in experiment 3 and was related to temperature and internal pressure changes (Figure 2a and Figure 3a). The initial pressure of 0.4 bar and 0.8 bar (experiment 2 and 4) produced lower rate of carbon dioxide than the initial pressure of 0.6 bar (experiment 3). This confirmed the previous conclusion that the initial pressure of 0.6 bar was preferred for composting OFMSW in Morocco. It was noticed that the profile of generated carbon dioxide was similar to that of the temperature. This indicated that the change of temperature was closely related to the change of carbon dioxide rate, suggesting that carbon dioxide rate might be used as another indicator for measuring the composting process besides temperature. The lowest emission of carbon dioxide was observed in experiment 1 and 5 which could be explained by its temperature profile. The low temperature is a sign of lower composting rate.

### Main study (initial MC effect)

#### Evolution of internal pressure

After determining the initial P_op_ that provides the required air amount for efficient composting, the second phase is devoted to the study of initial MC influence on composting process and on final composts quality. Subsequently, to determining the MC range and optimal moisture content (MC_op_) that allows efficient composting of OFMSW. Figure 2b shows the evolution of internal pressure change, ΔP, for the five experiments in this phase. As in the preliminary study, the change in internal pressure is an indicator of microbial activity in the feedstock. This variation was more pronounced in the first experiment of the second phase (85% humidity) with 1.6 bar, but dropped since the second day of composting. The maximum value of this variation can be explained by easily volatile compounds decomposition, while the rapid fall is due to humidity excess that has stifled the bio-respiration and inhibited the degradation process. In second and third experiment, changes in internal pressure were remarkable and they have reached their maximum of 1.3 bar and 1.2 bar respectively. The evolution of ΔP in these experiments was almost the same shape with a slight preference conditions in the second experiment that allowed more organic matter degradation into biogas. Moreover, the conditions in the fourth and fifth experiment were not favorable for composting process because the feedstock was relatively dry which involve low and slow organic matter degradation.

#### Temperature profile

After the initial filling of bioreactors, a rapid increase in temperature was produced in all experiments, indicating a marked microbial activity. The change in compost temperature followed a similar trend in typical composting process. Initially, the temperature in the feedstock increased as a result of the rapid breakdown of the readily available organic matter and nitrogenous compounds by micro-organisms (thermophilic phase). As the organic matter has become more stable, microbial activity, organic matter decomposition rate, and temperature gradually decreased to ambient levels. Temperature profiles of bioreactors containing different mixtures in the five experiments are shown in Figure 3b. The term ″optimum moisture″ is a compromise between the moisture requirements of micro-organisms and their simultaneous need for adequate oxygen supply [5]. Too high MC may inhibit the start of the composting process. Indeed, it′s the case of the first experiment of the second phase with initial moisture of 85%. In this study, MC in the range 70-75% was achieved at higher temperatures and keeps them longer. This provided the best conditions for disinfection and complete pathogens destruction. The temperature has reached its maximum values of 47°C and 46°C in the second and third experiment respectively in the second and the third day of composting. For the other three experiments, the maximum temperature has not exceeded 35°C. Thus, the proposed initial MC may be considered unfavorable for the composting process.

#### Evolution of pH and EC

**Figure 5:**
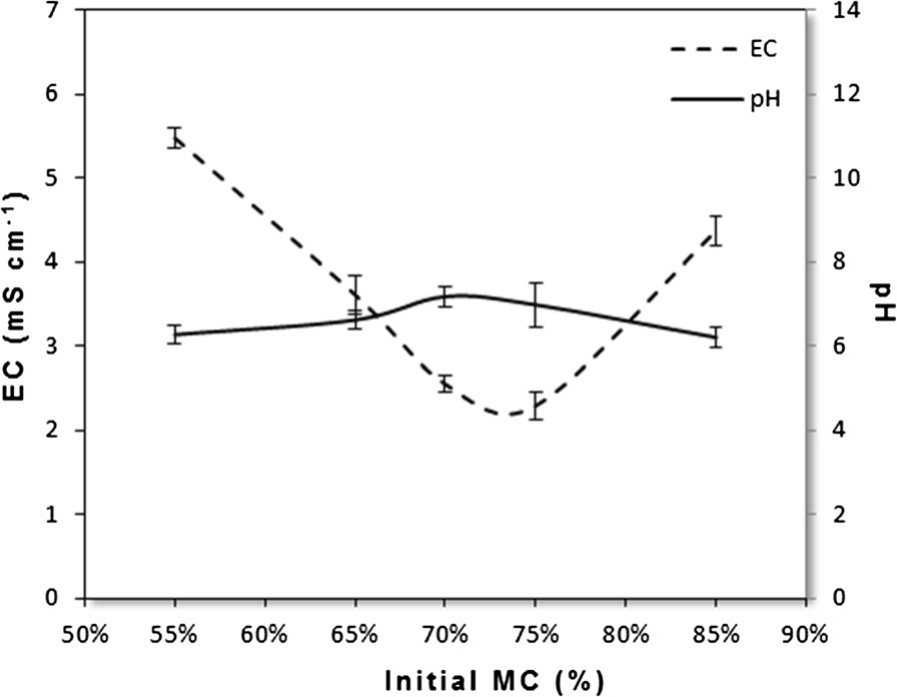
Changes of pH and EC for different experiments in the main study.

Measurements of pH and EC were performed before and at the end of each experiment. The Figure 5 show that pH varies very slightly (6.18 ≤ pH ≤ 7.18), indicating a good quality compost and within the suggested range of 6 – 8.5 as has been reported by several studies [25]. Moreover, we note in general a decrease from the initial conductivity of 7.2 to about 2.3 mS/cm in experiment 2 which indicate a decrease of 68%, while it has been dropped from 7.4 to 2.5 mS/cm in the experiment 3 (a decrease of 65%). Avnimelech *et al*. [26] found that the EC was initially 7.5 mS/cm and dropped after composting to a stable level of about 4 mS/cm. For organic waste, Corti *et al*. [27] and Erhart & Burian [28] reported that the EC of composts ranged between 0.14 and 12.2 mS/cm. In experiment 3, the EC of composting product did not exceed the limit content of 3 mS/cm, which indicated that EC would not adversely affect the plant growth [29].

#### Evolution of *NH*_4_^+^ − *N* and C/N ratio

**Figure 6:**
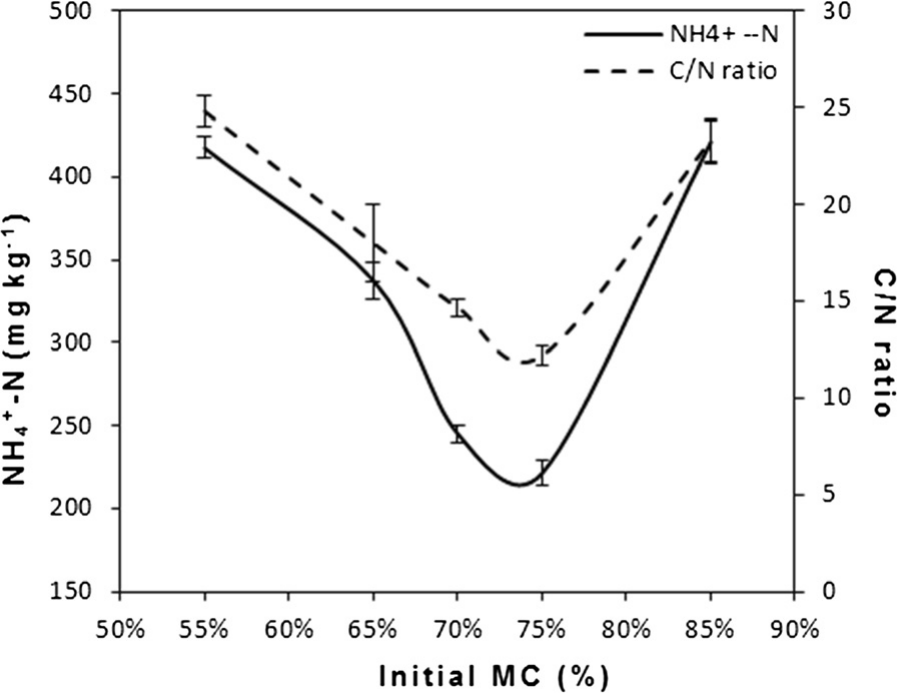
**Changes of *NH***_**4**_^**+**^ **−** ***N *and C/N ratio for different experiments in the main study.**

The *NH*_4_^+^ − *N* content in starting material was 524 mg/kg. Results of Figure 6 shows that the *NH*_4_^+^ − *N* content in final composts from the 5 experiments decreased to 421, 221, 245, 337 and 417 mg/kg respectively in the first, second, third, fourth and fifth experiment, due to the assimilation process carried out by micro-organisms, volatilization, and nitrification [30]. Riffaldi *et al.*[31] reported that the decrease in *NH*_4_^+^ − *N* was an indicator of both good composting and maturation process. Moreover, Zucconi and De-Bertoldi [32] recommended an *NH*_4_^+^ − *N* content of 400 mg/kg as the maximum content in mature compost. Thus the second, third and fourth compost meet the demand for agricultural applications.

Due to mineralization of organic matter, the C/N ratio in the five experiments decreased during composting process (Figure 6). The C/N ratio for all experiments decreased from initial value of about 26 respectively to 23.3, 12.2, 14.6, 18 and 24.8. However, when the initial C/N ratio is between 25 and 30, the final C/N ratio equals to or less than 20 is the standard for mature compost [33]. Therefore, the second, third and fourth compost has reached maturity according to this C/N criterion.

## Discussion

Composting is an aerobic process; a supply of air to the feedstock is needed to provide oxygen for decomposer micro-organisms and to drive produced gases by biodegradation. To maintain good oxygenation, ventilation and turning are important. They will mix the materials in order to make it easily accessible and be attacked by microbial flora and also to maintain ventilation often blocked by the compaction phenomenon. Thereafter, the air renew and turning allow restarting the biological process and increase the temperature again, which explains sudden temperature changes observed during different phases (preliminary and main studies). In our experiments, the air renews and turnings were made every day and produced gases were then evacuated at the end of the day.

According to the preliminary study, initial air pressure in the bioreactor affects temperature evolution and gas production. For initial air pressures equal or less than 0.4 bar, oxygen amount inside the bioreactor is insufficient to complete organic matter degradation, and it quickly runs out and prevents better composting process conduction. Therefore, the temperature rose slightly but did not reach significant levels. We also note low CO_2_ emissions in benefit of other gases that are, possibly, anaerobic degradation products. For initial air pressures equal or more than 0.8 bar, the feedstock oxygenation is better but we note always low levels either in temperature or CO_2_ production. This can be explained by the high pressure that destroys micro-organisms responsible for organic matter degradation. Furthermore, an initial air pressure above 0.6 bar provides a suitable environment for microorganisms′ growth giving higher temperature levels and remarkable carbon dioxide production.

In the main study, the observed increase in temperatures is the result of organic matter oxidation by aerobic microbial population. This activity is important at the beginning of composting process; it is reflected in the increase of temperature. Thus, temperature monitoring allows an indirect measure of aerobic degradation intensity. At temperatures below 20°C, only psychrotrophic micro-organisms are active. Between 20 and 40°C, it is the turn of mesophilic ones, but thermophilic micro-organisms are active only at temperatures between 40 and 70°C [34].

At beginning of composting, during the first day, biological activity settles through mesophilic micro-organisms. This activity quickly raises the temperature in experiment 2 and 3, with dominance of bacteria that degrade easily degradable compounds such as sugars, proteins and lipids [35]. The temperature evolution during composting will depend on fermented substrate decomposition, and calorific value. Food scarps are high in fermentable compounds, since temperatures in the range of 40 – 50°C are rapidly achieved.

After that, thermophilic phase is taking place (Figure 3b) and takes about 5 days. Actinomycetes, thermophilic fungi [36,37] and thermophilic bacteria [38,39] are most active during this phase of composting. They are the most anticipated for two main reasons: they speed up the decomposition process and ensure compost sanitation by removing pathogens carried by various waste compounds. The actinomycetes and thermophilic fungi break down complex carbon sources such as cellulose and hemicellulose [34,36].

But for high temperatures, activity of the heat-sensitive micro-organisms which participate in the compost maturation is inhibited. These temperatures become limiting for biological activity, which reduces the amount of generated heat, and the temperature stabilizes until conditions become limiting, particularly the substrate decomposition, which leads to a gradual decrease in temperature. This cooling phase lasts approximately 4 days in the second and the third experiment; the temperatures reach values from 27 to 25°C that are close to ambient after 8 days of composting (Figure 3b). At this stage, the maturation phase begins and the mesophilic flora relocates to end cellulose and lignin degradation [40,41].

The organic matter was mineralized after composting, mainly due to the degradation of easily degradable compounds, which are used by microorganisms as a source of carbon and nitrogen. To degrade organic compounds, micro-organisms convert 60 - 70% carbon dioxide and use 30 - 40% remaining in their bodies as cellular components [42]. The rate of organic matter degradation is an indicator of the overall composting rate. The smallest organic matter degradation was carried out in experiment 4 and 5, which had the lowest initial MC (65% and 55%). Moreover, the initial moisture 85% inhibited the composting process, and therefore it has also degraded a small amount of organic matter. The greatest degradation was done by experiment 2 and 3, which had MC in the range 70 - 75%.

The results indicated that initial MC directly affected the total carbon dioxide production and therefore, organic matter biodegradation. Indeed, microbial growth depends on MC because water, for example, is a reactant during hydrolysis. Nevertheless, MC must not limit the transfer of gases such as O_2_ and CO_2_. By acting on permeability, FAS influences airflow pathways and thereby biodegradation kinetics through heat and gas transfers [43]. The results show that increasing MC to an optimum level improved organic matter biodegradation.

Beyond this optimum MC, water reduced aeration and microbial O_2_ supply [44]. The organic matter content of the materials decreased in all experiments during the process, but this reduction was greater in experiment 2 than others (Table 2). This suggested that the higher MC probably enhanced the biodegradation of the composting material, which could be further verified by the higher temperature attained and more production of carbon dioxide in material with higher MC. The results with organic matter degradation verified that initial MC of 75% seems to be favorable for composting the OFMSW.

**Table 2:** **Conversion of organic matter** (**k**) **at the end of the composting process**

**Experiment**	**1**	**2**	**3**	**4**	**5**
**k** (%)	14.66	31.61	29.21	26.75	11.57

## Conclusion

The results suggest that initial MC in the range 70 - 75% under 0.6 bar pressure can be considered suitable for efficient further composting OFMSW in Morocco. Composting is a very complex biologically based process and it is influenced by a number of factors (temperature, aeration, MC, particle size, carbon to nitrogen ratio, pH, porosity, etc.). These factors are very inter-related and therefore they can have direct and indirect effects. Thus, the MC of 75% may be nearer to the optimum than the others. It would be wrong to suggest that it was the optimum without having some kind of proof. Therefore, pilot-scale and full-scale experiments, using the same initial MC, need to be performed in order to confirm this finding.

There is no universally applicable optimum MC for composting materials. This is because each material has unique physical, chemical and biological characteristics, and these affect the relationship between MC and its corollary factors water availability, particle size, porosity, and permeability.

## Competing interests

The authors declare that they have no competing interests.

## Authors’ contributions

AM has designed and performed experiments, analyzed data and wrote the manuscript. OA has guide in the manuscript preparation. MM has guided in the field works, interpretation techniques as well as manuscript preparation. All authors read and approved the final manuscript.
